# Autophagy modulates temozolomide-induced cell death in alveolar Rhabdomyosarcoma cells

**DOI:** 10.1038/s41420-018-0115-9

**Published:** 2018-10-25

**Authors:** Adel Rezaei Moghadam, Simone C. da Silva Rosa, Ehsan Samiei, Javad Alizadeh, Jared Field, Philip Kawalec, James Thliveris, Mohsen Akbari, Saeid Ghavami, Joseph W. Gordon

**Affiliations:** 1 Department of Human Anatomy and Cell Science, Max Rady College of Medicine, Rady Faculty of Health Science, University of Manitoba, Winnipeg, Canada; 2 Laboratory for Innovations in Microengineering (LiME), Department of Mechanical Engineering, University of Victoria, Victoria, BC Canada; 3 Health Policy Research Centre, Shiraz University of Medical Sciences, Shiraz, Iran; 4 Colleges of Medicine and Nursing, Rady Faculty of Health Science, University of Manitoba, Winnipeg, Canada; 5 Center for Biomedical Research, University of Victoria, Victoria, Canada; Center for Advanced Materials and Related Technology (CAMTEC), University of Victoria, Victoria, Canada; 6 The Biology of Breathing Theme, Children’s Hospital Research Institute of Manitoba, University of Manitoba, Winnipeg, Canada; 7 The Diabetes Research Envisioned and Accomplished in Manitoba (DREAM) Theme, Children’s Hospital Research Institute of Manitoba, University of Manitoba, Winnipeg, Canada

**Keywords:** Sarcoma, Macroautophagy, Apoptosis

## Abstract

Rhabdomyosarcoma (RMS) is a muscle-derived tumor. In both pre-clinical and clinical studies Temozolomide (TMZ) has been recently tested against RMS; however, the precise mechanism of action of TMZ in RMS remains unclear. Here we demonstrate that TMZ decreases the cell viability of the RH30 RMS and C2C12 cell line, where cells display evidence of mitochondrial outer membrane permeability. Interestingly, the C2C12 mouse myoblast line was relatively more resistant to TMZ-induced apoptosis. Moreover, we observed that TMZ activated biochemical and morphological markers of autophagy in both cell lines. Autophagy inhibition in both RH30 and C2C12 cells significantly increased TMZ-induced cell death. In RH30 cells, TMZ increased Mcl-1 and Bax protein expression compared to corresponding time match controls while in C2C12 Mcl-1, Bcl-2, Bcl-XL, and Bax protein expression were not changed. Baf-A1 co-treatment with TMZ significantly decrease Mcl-1 expression compared to TMZ while increase Bax expression in C2C12 cells (Bcl2 and Bcl-XL do not significantly change in Baf-A1/TMZ co-treatment). Using a three-dimensional (3D) C2C12 and RH30 culture model we demonstrated that TMZ is significantly more toxic in RH30 cells (live/dead assay). Additionally, we have observed in our 3D culture model that TMZ induced both apoptosis (cleavage of PARP) and autophagy (LC3-puncta and localization of LC3/p62). Therefore, our data demonstrate that TMZ induces simultaneous autophagy and apoptosis in both RH30 and C2C12 cells in 2D and 3D culture model, where RH30 cells are more sensitive to TMZ-induced death. Furthermore, autophagy serves to protect RH30 cells from TMZ-induced death.

## Introduction

Rhabdomyosarcoma (RMS) is an aggressive soft-tissue malignant tumor that occurs in both children and adults^1^, but comprises up to 50% of all childhood soft tissue sarcomas^2,3^. Clinically, survival among patients with metastatic RMS has not improved appreciably in the past years, emphasizing an urgent need to develop new strategies to treat and prevent this disease^4^. Four subgroups of RMS have been described based on histological, genetic, and clinical criteria^5^: embryonal RMS, pleomorphic RMS, spindle cell/sclerosing RMS, and alveolar RMS (ARMS). ARMS is an aggressive subtype of RMS suffered by adolescents and young adults^5^. In addition, the high-mortality rate in ARMS has been attributed to the presence of oncogenic fusion proteins (i.e., PAX3-FOXO1 and PAX7-FOXO1) generated by chromosomal translocations^6^.

Recently, oral alkylating agents such as TMZ have received considerable attention in RMS pre-clinical and clinical studies^7^. TMZ has a broad spectrum of antitumor activity while being well-tolerated by the patient due to its relatively low toxicity^8–10^. The mechanism of action of TMZ results in the production of a highly reactive methyldiazonium cation^11^ that transfers its methyl group to purine bases of DNA resulting in double-stranded breaks during repair^12–15^. This process leads to G2/M cell cycle arrest and activation of apoptosis^16–18^. However, the cellular response to TMZ also involves alterations in gene expression that have been shown to be cancer-cell specific. Thus, the pathways involved in apoptosis induction may be different for each type of sarcoma, and there is little information regarding how TMZ affects ARMS at the cellular level. In our studies, we explored the role of autophagy in RH30 cells to further elucidate the mechanism of action of TMZ.

Autophagy is a conserved physiological process of cellular self-eating, which plays an essential role in cellular housekeeping activity by degrading protein aggregates, cytoplasmic components, and damaged or dysfunctional organelles. At least three distinct forms of autophagy can be activated depending on the route that cytoplasmic material is delivered to lysosomes, such as chaperone-mediated autophagy, microautophagy, and macroautophagy (from here on referred to as autophagy)^19–22^. The role of autophagy in cancer cell biology is complicated and evolves throughout tumorigenesis. For instance, autophagy has been shown to promote cancer cell survival during conditions of a nutrient or hypoxic stress and contribute to cell demise through autophagic cell death (i.e., type II programmed cell death)^23^. More recently, autophagy has also been shown to contribute to epithelium to mesenchymal transition (EMT) and promote cancer metastasis in different cancer models^21,24^. In RH30 cells, autophagy is known to be a crucial process in the maintenance of cellular viability and proliferation^25^. Furthermore, inhibition of autophagy by the Atg7 knockdown, or pharmacological inhibition with chloroquine or Baf-A1 treatment, has been demonstrated to decrease cell growth and reduced viability in RMS cell lines^26,27^.

Autophagy and apoptosis are two independent processes, but under certain conditions, they cooperate in a hierarchical relationship to regulate the turnover of organelles and proteins within cells, and of cells within organisms^28,29^. However, within a given cell, considerable cross-talk exists between apoptosis and autophagy, and nature of this cross-talk can change in a cell context-dependent manner^30–32^. Generally, autophagy is a rapidly induced survival pathway activated by sublethal stress, whereas apoptosis is initiated at lethal doses of stress^30^. However, in certain conditions, autophagy may also contribute to the induction of cell death by either the activation of programmed cell death type II, activation of mitochondrial-dependent cell death pathways (i.e. apoptosis or necrosis), or by providing substrates to promote ATP-dependent apoptotic mechanisms^33–35^. The BCL-2 family of proteins plays a crucial role in the regulation of the cross-talk between apoptosis and autophagy, consisting of both pro-survival and pro-death family members^36,37^. However, the role of the BCL-2 family in regulating apoptosis and autophagy in ARMS is not well dissected.

In this report, we demonstrate that TMZ decreased cell viability of the RH30 RMS cell line and C2C12 cell line in presence of autophagy activation. TMZ induces outer mitochondrial membrane permeabilization without changes in mitochondrial membrane potential. We also show that treatment of RH30 and C2C12 cells with low concentrations of the autophagy flux inhibitor Baf-A1, significantly increased TMZ-induced cell death. Collectively, these observations help define the role of autophagy in RH30 cells and contribute to our knowledge regarding the mechanism of action of TMZ in PAX3-FOXO1 positive sarcomas.

## Results

### TMZ induces apoptotic cell death in C2C12 and RH30 cell lines

**Fig. 1:**
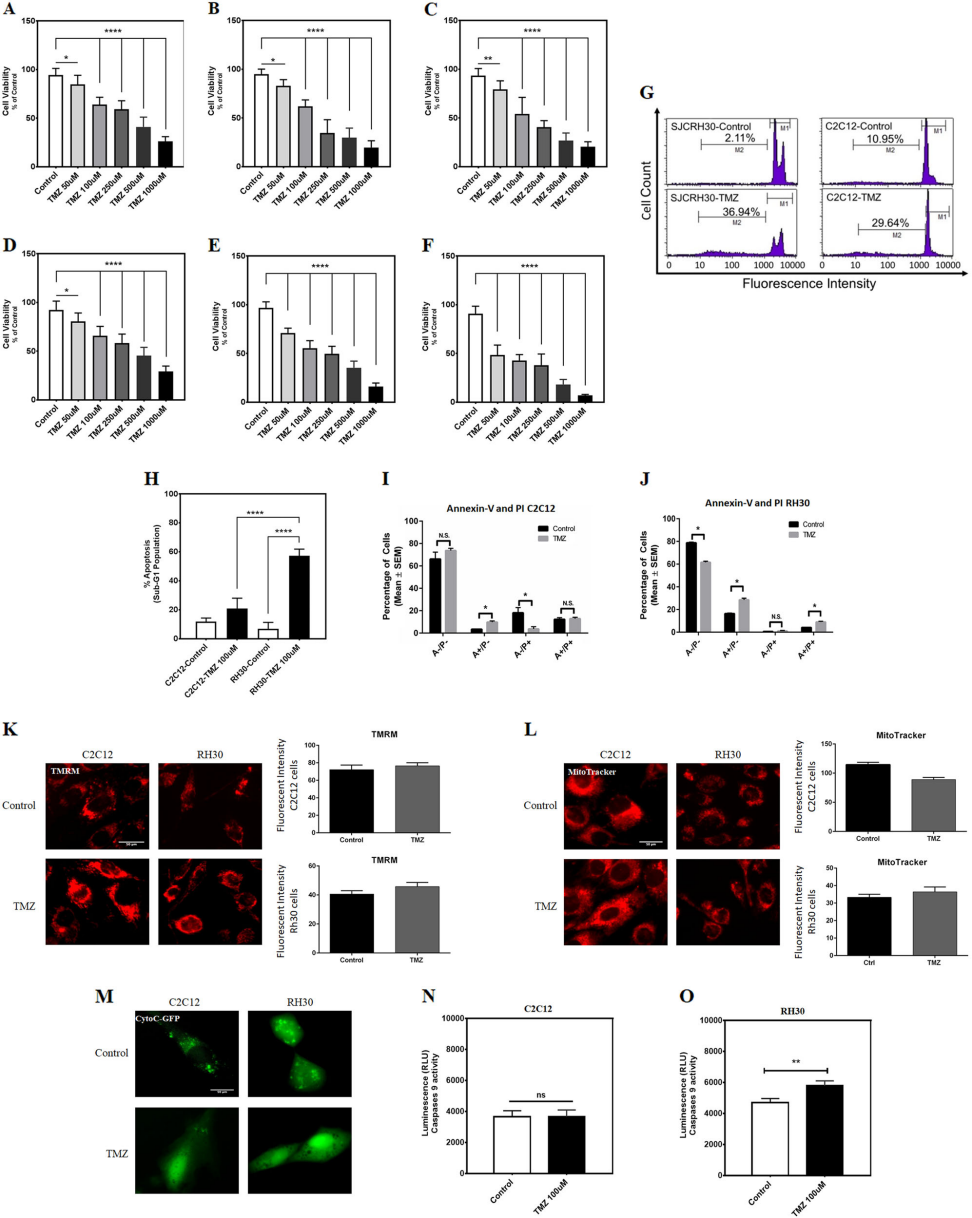
TMZ induces apoptotic cell death in C2C12 and RH30 cell lines. **a**–**c** C2C12 cells were treated with TMZ (50, 100, 250, and 1000 μM) and cell viability was assessed 48, 72, and 96 h after that by MTT assay. Control cells for each time point were treated with the solvent control (DMSO). Results are expressed as a percentage of corresponding time point control and represent the means ± SD of 15 replicates in three independent experiments (**P < 0.01; ***P < 0.0001). **d**–**f** C2C12 cells were treated TMZ (50, 100, 250, and 1000 μM) and cell viability was assessed 48, 72, and 96 h after that by MTT assay. Control cells for each time point were treated with the solvent control (DMSO). Results are expressed as a percentage of corresponding time point control and represent the means ± SD of 15 replicates in three independent experiments (**P < 0.01; ****P < 0.0001). **g** Representative figures of the flow cytometry histogram for C2C12 and RH30 are shown. Cells were treated with TMZ (100 μM, 72 h) and Percent sub-G1 abundance induced by TMZ (100 μM) or DMSO solvent control after 72 h. The Sub-G1 population showed an abundance of apoptotic cell death in each treatment. **h** The average of the sub-G1 population of C2C12 and RH30 cells which were treated with TMZ (100 μM, 72 h) and DMSO solvent control has been measured. Results represent the means ± SD of six replicates in three independent experiments (****P < 0.0001). **i**, **j** C2C12 and RH30 cells were treated with TMZ and stained with Annexin-V-FITC (A) and PI (P) and analyzed by flow cytometry (*n* = 3)(*P < 0.05). **k** TMRM staining of C2C12 and RH30 cells treated with TMZ (100 μM, 48 h). **l** MitoTracker staining of C2C12 and RH30 cells treated with TMZ (100 μM, 48 h). **m** C2C12 and RH30 cells were transfected with cytochrome C-GFP (CytoC-GFP) and treated with TMZ (100 μM, 48 h)(*P < 0.05). **n**, **o** C2C12 and RH30 cells were treated with 100 μM TMZ for 48 h and then Caspase-9 activity was measured using Caspase-Glo luminescence assay. (**P < 0.01)

We treated both the RH30 RMS cell line and C2C12 myoblasts with TMZ (0, 50, 100, 250, 500, and 1000 μM in 48, 72, and 96 h) and cell viability was examined by MTT assay. TMZ-induced cell death in a dose- and time-dependent manner in C2C12 cells (Fig. 1a–c) and RH30 cells (Fig. 1d–f), where RH30 cells appeared more sensitive to the effects of TMZ. We confirmed that TMZ (100 μM, 72 h) increases apoptotic cell death in both C2C12 and RH30 cell lines using a propidium iodide (PI)-dependent method (i.e. Nicoletti assay). Following TMZ treatment, the percentage of apoptotic cells was approximately three times more than control C2C12 cells (Fig. 1g). However, in RH30 cells, TMZ-induced apoptosis by 17.5 times compared to the control cells (Fig. 1g). Therefore, TMZ significantly activated higher levels of apoptosis in RH30 cells than in C2C12 cells (Fig. 1h). We also performed flow cytometry analysis where cells were stained with Annexin-V and PI (Fig. 1i, j). In C2C12 cells treated with TMZ, we observed an increase in Annexin-V positive (A+/P−) cells, but no change in double positive cells (A+/P+). Interestingly, we also observed a decrease in PI positive cells (A−/P+) (Fig. 1i). Conversely, in RH30 cells treated with TMZ we observed significant increases in Annexin-V positive and double positive cells, with a corresponding decreased in double negative cells (A−/P−) (Fig. 1j). These observations are consistent with our MMT and Nicoletti findings, and suggest that RH30 cells are more sensitive to the apoptosis inducing effects of TMZ compared to C2C12 cells.

To investigate the cellular mechanisms behind the differential response to TMZ treatment, we performed a series of experiments to evaluate intrinsic cell death pathways initiated by mitochondria. We stained cells with the mitochondrial membrane potential-sensitive dye TMRM^38,39^, as rapid dissipation of mitochondrial membrane potential has been recently associated with mitochondrial permeability transition. Surprisingly, we observed that TMZ treatment did not lead to mitochondrial depolarization at this dose and time in either cell line (Fig. 1k). No change in MitoTracker staining was observed in RH30 cells, indicating that mitochondrial content was not altered by TMZ treatment, however we did observe a modest, but significant reduction in MitoTracker staining in C2C12 cells (Fig. 1l). Next, we transfected cells with a cytochrome C-GFP fusion construct that has been previously shown to accumulate in the mitochondrial intermembrane space and be released into the cytosol during mitochondrial outer membrane permeabilization (MOMP)^40^. Following transfection, we observed fluorescent mitochondrial puncta in both untreated cell lines (Fig. 1m). When RH30 and C2C12 cells were treated with TMZ, we observed release of cytochrome C-GFP into the cytosol. In RH30 cells, no fluorescent puncta remained following TMZ treatment; however, in C2C12 cells residual puncta remained in up to 20% of cells (Fig. 1m). Collectively, these findings indicate that TMZ induces MOMP without mitochondrial permeability transition.

Caspase-9 is an important effector of intrinsic or mitochondrial-regulated apoptosis^41–44^. It has been recently reported that TMZ induces caspase-dependent apoptosis in several cancer cells^45,46^. Thus, we determined the effects of TMZ-induced MOMP on caspase-9 activation. Following treatment of TMZ 100 μM for 48 h (Fig. 1n, o), we observed a significant activation of caspase-9 in RH30 cells (**P < 0.01); however, the caspase-9 activity was unchanged in C2C12s at this dose and time (*P* > 0.05).

### TMZ induces autophagy activation in C2C12 and RH30 cells

**Fig. 2:**
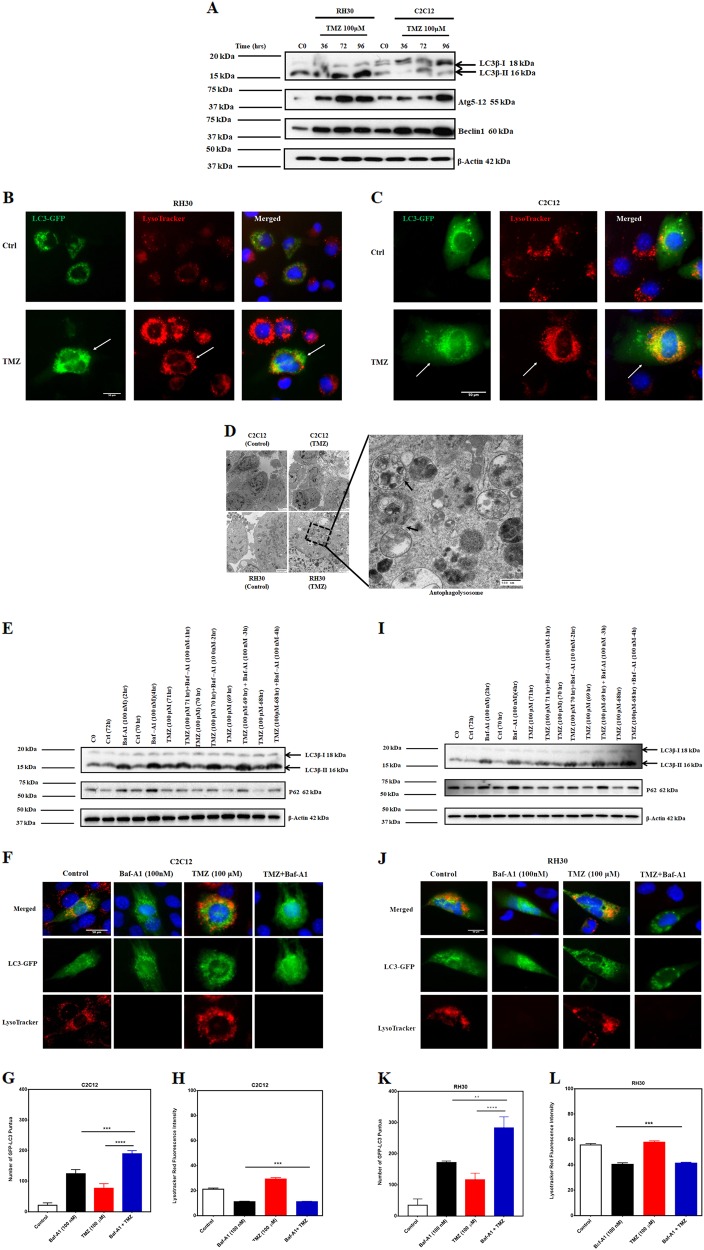
TMZ induces autophagy in C2C12 and RH30 cell lines. **a** C2C12 and RH30 cells were treated with TMZ (100 μM, 36, 72, 96 h) and autophagy hallmark was detected in both C2C12 and RH30 cell lines using immunoblotting. TMZ-induced LC3β lipidation, Atg5-12 conjugation, and Beclin-1 expression in both cell lines. Beta-actin was used as loading control. Data are representative of three independent experiments using different cultures. **b**, **c** RH30 and C2C12 cells were treated with TMZ (100 μM, 72 h) and using immunocytochemistry LC3 puncta and changes in lysosomal activity (LysoTracker red staining) has been investigated. The results showed that TMZ increased LC3 puncta and LysoTracker red fluorescence intensity and co-localization of LC3 puncta and LysoTracker in both RH30 and C2C12 cells. **d** Transmission electron microscopy showed that in treated RH30 and C2C12 cells there are accumulated autophagosome-like structures compared to control and normal cells after 72 h treatment. Arrows show the autophagolysosomes containing the cargo (magnification ×11,600). **e** C2C12 cells were treated with TMZ (100 µM, 72 h) and then treated with Baf-A1 (100 nM, 1, 2, 3, 4 h) to evaluate for autophagy flux. TMZ + Baf-A1 treatment induces more lipidated LC3β and reduces the degradation of p62 in RH30 cells. Beta-actin was used as the loading control. Data are representative of three independent experiments. **f** C2C12 cells were treated with TMZ (100 μM, 72 h) and Baf-A1 (100 nM, +4 h) followed by immunocytochemistry to evaluate LC3 puncta and changes in lysosomal activity (LysoTracker red staining). The results showed that TMZ increased LC3 puncta and LysoTracker red fluorescence intensity in C2C12 cells. On the other hand, Baf-A1 and TMZ + Baf-A1 significantly decreased the LysoTracker red fluorescence in the presence of an accumulation of LC3 puncta showing the inhibition of autophagy flux by Baf-A1. **g**, **h** Representative figures of LC3 puncta and fluorescence intensity for LysoTracker in C2C12 cells in the presence of TMZ, Baf-A1, and TMZ/Baf-A1 treatment. These results showed that the number of LC3 puncta is significantly higher in cells which are treated with Baf-A1 and TMZ + Baf-A1. However, the fluorescence intensity of LysoTracker was lower in Baf-A1, and TMZ + Baf-A1 treated cells. **i** RH30 cells were treated with TMZ (100 µM, 72 h) and then treated with Baf-A1 (100 nM, 1, 2, 3, 4 h) to evaluate for autophagy flux. TMZ/Baf-A1 treatment induces more LC3β lipidation and reduces the degradation of p62 in RH30 cells compared to TMZ and Baf-A1 single treatment. Beta-actin was used as the loading control. Data are representative of three independent experiments. **j** RH30 cells were treated with TMZ (100 μM, 72 h) and Baf-A1 (100 nM, +4 h) followed by immunocytochemistry to evaluate LC3 puncta and changes in lysosomal activity (LysoTracker red staining). The results showed that TMZ increased LC3 puncta and LysoTracker fluorescence intensity in RH30 cells. On the other hand, Baf-A1 and TMZ + Baf-A1 significantly decreased the LysoTracker red fluorescence showing the inhibition of autophagy by Baf-A1. **k**, **l** Representative figures of LC3 puncta and fluorescence intensity for LysoTracker in RH30 cells in the presence of TMZ, Baf-A1, and TMZ/Baf-A1 treatment. These results showed that many LC3 puncta are significantly higher in cells which are treated with Baf-A1 and TMZ + Baf-A1. However, the fluorescence intensity of LysoTracker was lower in Baf-A1, and TMZ + Baf-A1 treated cells

Previous reports have shown that TMZ induces simultaneous apoptosis and autophagy in glioblastoma cells^22,47^. Thus, we evaluated if differential activation of autophagy could explain the increased toxicity to TMZ in RH30 cells compared to C2C12 cells. Our results demonstrate that TMZ induces autophagy in both C2C12 and RH30 cell lines. Immunoblotting results showed lipidation of LC3B, Atg5-12 conjugation, and increase in Beclin-1 expression in both cell lines, indicating autophagy activation by TMZ (100 µM) (Fig. 2a). In RH30 and C2C12 cells, fluorescent microscopy confirmed that TMZ increased the number of LC3-GFP puncta and lysosomal activation (LysoTracker red fluorescence intensity), which co-localized in TMZ treated cells (Fig. 2b, c). Also, transmission electron microscopy (TEM) further confirmed autophagy activation in RH30 and C2C12 cells (double membrane autophagosome formation), after 72 h treatment with TMZ (100 µM) (Fig. 2d).

To investigate whether the accumulation of LC3B-II is due to an increase in autophagic flux or prevention of autophagic proteolysis, we used Baf-A1 (100 nM) for different time points (2, 3, 4 h) to study autophagy flux. It has been previously shown that a high concentration of Baf-A1 with short time point exposures decreases lysosome numbers, causes fusion block, and suppresses autolysosome destruction by interfering with late-stage autophagosome-lysosome fusion^19,48^. As shown by western blot, treatment with Baf-A1 (100 nM) 2, and 4 h markedly increased the accumulation levels of LC3-II and P62 in both C2C12 and RH30 cells. Combination treatment of cells with TMZ (100 µM, 72 h) and Baf-A1 (100 nM) at 1, 2, 3, and 4 h showed a time-dependent accumulation of LC3B-II compared to cells treated with Baf-A1 (100 nM) alone (C2C12 cells Fig. 2e; RH30 cellsFig. 2i). These results indicate that TMZ induces autophagy flux in both cell lines. Consistent results were obtained using GFP-LC3, where in cells treated with a combination of TMZ and Baf-A1 (3 h, 100 nM) we observed increased GFP-LC3 puncta, compared to cells treated with Baf-A1 (100 nM) alone (C2C12 cells Fig. 2e; RH30 cells Fig. 2i). Lysosomal activity was found to be lower in cells after treatment with Baf-A1 (100 nM) alone, as well in combination with TMZ (100 µM, 60 h) compared to the control and TMZ alone group, confirming lysosomal acidification inhibition and blockage of the autophagy flux (C2C12 cells Fig. 2f–h; RH30 cells Fig. 2i–l). Moreover, we demonstrate that the number of autophagic C2C12 cells is higher in cells treated with Baf-A1 with and without TMZ. However, the fluorescence intensity of LysoTracker was lower in Baf-A1 and in TMZ + Baf-A1 treated cells (C2C12 cells Fig. 2f–h; RH30 cells Fig. 2i–l). Overall, these findings suggest that TMZ induces autophagy flux in C2C12 and RH30 cells.

### Autophagy inhibition increases TMZ-induced apoptosis in C2C12 and RH30

**Fig. 3:**
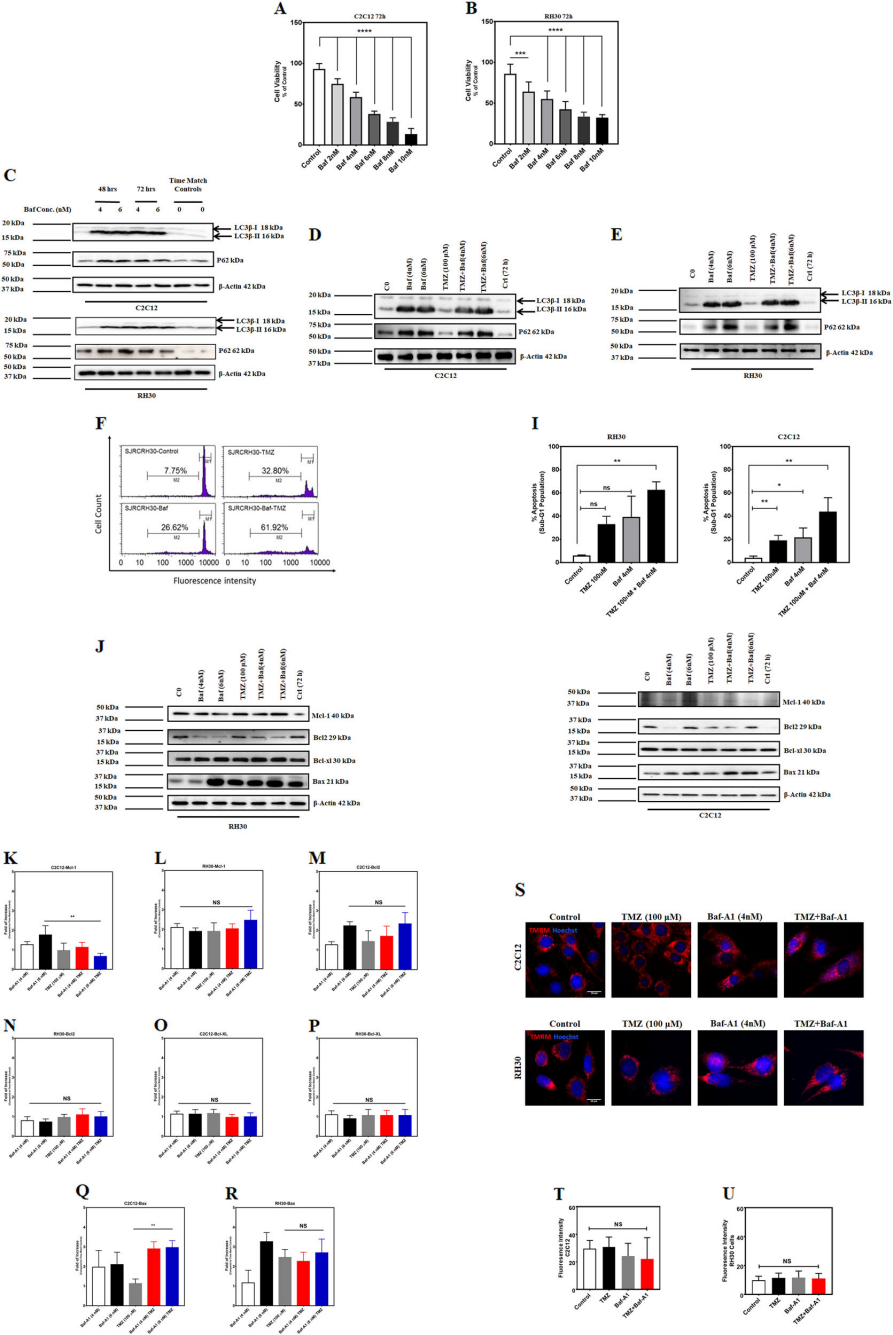
Autophagy inhibition increases TMZ-induced apoptosis in C2C12 and RH30 cells. **a**, **b** C2C12 and RH30 cells were treated with Baf-A1 (2, 4, 6, 8, and 10 nM) and cell viability was assessed after 72 h using MTT assay. Control cells for each time point were treated with the solvent control (DMSO). Results are expressed as a percentage of corresponding time point control and represent the means ± SD of 15 replicates in three independent experiments (**P < 0.01; ***P < 0.0001). **c** C2C12 and RH30 cells were treated with Baf-A1 (4 and 6 nM, 48, 72 h) and autophagy hallmark proteins were detected in both C2C12 and RH30 cell lines using immunoblotting. Baf-A1 induces accumulation of lipidated LC3β and decreases the degradation of p62 in both cell lines. Beta-actin was used as loading control. Data are representative of three independent experiments using different cultures. **d**, **e** C2C12 and RH30 cells were treated with TMZ (100 µM, 72 h) and then treated with Baf-A1 (4, 6 nM, 72 h) to evaluate autophagy inhibition in the presence of Baf-A1 treatment. TMZ + Baf-A1 treatment induces LC3β lipidation and reduces the degradation of p62 in C2C12 and RH30 cells. Beta-actin was used as the loading control. Data are representative of three independent experiments. **f**–**h** Representative figures of the flow cytometry histogram for RH30 are shown. Cells were treated with TMZ (100 μM, 72 h), Baf-A1 (4 nM, 72 h) and Baf-A1/TMZ. Percentage of sub-G1 abundance induced by TMZ (100 μM, 72 h), Baf-A1 (4 nM, 72 h) and Baf-A1/TMZ after 72 h has been showed. The Sub-G1 population showed an abundance of apoptotic cell death in each treatment. **g**, **h** C2C12 and RH30 cells were treated with TMZ (100 µM, 72 h) and Baf-A1 (4 nM, 72 h) and then cell lysates were collected to examine the effect of TMZ, Baf-A1, and TMZ/Baf-A treatment on expression of Bcl2 family proteins (Bcl-2, Bcl-XL, Mcl-1, and Bax). **k**–**r**, **k**, **l** TMZ does not significantly change Mcl-1 expression in both C2C12 and RH30 cells. It is notable that Baf-A1 (6 nM)/TMZ co-treatment significantly (*P* < 0.01) decrease Mcl-1 expression in C2C12 cells while does not have any effect in RH30 cells. **m**, **n** TMZ does not significantly change Bcl-2 expression in both C2C12 and RH30 cells. TMZ/Baf-A combination treatment also does not significantly change Bcl-XL expression in both C2C12 and RH30 cells. **o**, **p** TMZ does not significantly change Bcl-XL expression in both C2C12 and RH30 cells. TMZ/Baf-A combination treatment also does not significantly change Bcl-2 expression in both C2C12 and RH30 cells. **q**, **r** TMZ does not significantly change Bax expression in both C2C12 and RH30 cells. TMZ/Baf-A combination treatment significantly increase Bax-expression in C2C12 cells (*P* < 0.01) while does not have a significant effect on its expression in RH30 cells. **s**–**u** We have evaluated changes in mitochondrial membrane potential in the presence of TMZ (100 µM, 60 h), Baf-A1 (4 nM, 60 h), and TMZ/Baf-A1 combination in C2C12 and RH30 cells. **s** Representative images show the mitochondrial membrane potential measured by TMRM. Red color denotes TMRM staining. **t**, **u** Measurement of the mean of TMRM fluorescence intensity shows that TMZ (100 µM, 60 h), Baf-A1 (4 nM, 60 h), and TMZ/Baf-A1 combination does not change mitochondrial membrane potential in C2C12 **t** and RH30 **u** cells. The data are representative of the mean fluorescence in at least 100 cells in each cell type. The data were analyzed by Student’s *t*-test or ANOVA, followed by post hoc analysis. If *p* < 0.05, results were considered statistically significant

Next, we examined the effect of chemical inhibition of autophagy on TMZ-induced apoptosis. Previously, we demonstrated that low dose concentrations of Baf-A1 inhibits autophagy, but cell toxicity can occur at higher doses^49,50^. Therefore, we performed a cell viability assay (MTT) to establish the appropriate concentration of Baf-A1 to inhibit autophagy with the least cytotoxic effect on C2C12 and RH30 cells. C2C12 and RH30 cells were treated with different concentration of Baf-A1 (0.1–10 nM) for 48 and 72 h. Our results revealed that Baf-A1 (4 and 6 nM) had low cytotoxicity at 72 h in both cell lines (Fig. 3a, b), thus we evaluated the effectiveness of these two Baf-A1 concentrations to inhibit autophagy. Cells were treated with Baf-A1 (4 and 6 nM) for 48, and 72 h and autophagy markers (LC3 lipidation and P62 degradation/accumulation) were investigated in both cell lines using western blotting. We observed that Baf-A1 (4 and 6 nM) inhibits autophagy, determined by the accumulation of lipidated LC3B-II and P62 (Fig. 3c) in both C2C12 and RH30 cells. Next, we confirmed the effect of Baf-A1 (4 and 6 nM) on autophagy markers in C2C12 and RH30 cells treated with TMZ (100 µM/ml; Fig. 3d, e). Finally, we evaluated apoptosis by flow cytometry analysis with PI staining in cells treated with TMZ (100 μM) and Baf-A1 (4 nM) for 72 h. Both TMZ and Baf-A1 increased the apoptotic cell population compared to control; however, the percentage of apoptosis in cells treated with the combination of TMZ and Baf-A1 was greater than either TMZ or Baf-A1 alone (Fig. 3f–h). Collectively, these findings demonstrate that autophagy inhibition increases TMZ-induced apoptosis in both C2C12 and RH30 cells.

To further dissect the mechanisms by which TMZ-induces apoptosis in presence of autophagy inhibition, we evaluated the expression of Bcl2 family proteins by western blot in RH30 and C2C12 cells treated with TMZ and/or Baf-A1, as previously described^31,35–37^. We evaluated Bcl-2, Bcl-xl, Mcl-1 (anti-apoptotic), and Bax (pro-apoptotic). As shown in Fig. 3i, j, Baf-A1 (6 nM) co-treatment with TMZ (100 µM) significantly decreased Mcl-1 expression (*P* < 0.01) compared to TMZ (100 µM) with increased Bax expression (*P* < 0.01) in C2C12 cells. Bcl-2 and Bcl-XL did not significantly change in Baf-A1/TMZ co-treatment (Figure K, M, N, and Q). Interestingly, we did not see any significant changes in RH30 cells with regards to levels of Mcl-1, Bcl-2, Bcl-XL, or Bax expression after co-treatment of Baf-A1/TMZ (Fig. 3l, n, p, r). We also evaluated the effect of TMZ (100 µM, 60 h) and autophagy inhibition (Baf-A1, 4 nM, 60 h) on mitochondrial membrane potential in C2C12 and RH30 cells. Interestingly, the results showed that TMZ, Baf-A1, and TMZ/Baf-A co-treatment do not affect mitochondrial membrane potential in either C2C12 and RH30 cells (Fig. 3s–u).

### TMZ induces apoptosis and autophagy in C2C12 and RH30 3D culture

**Fig. 4:**
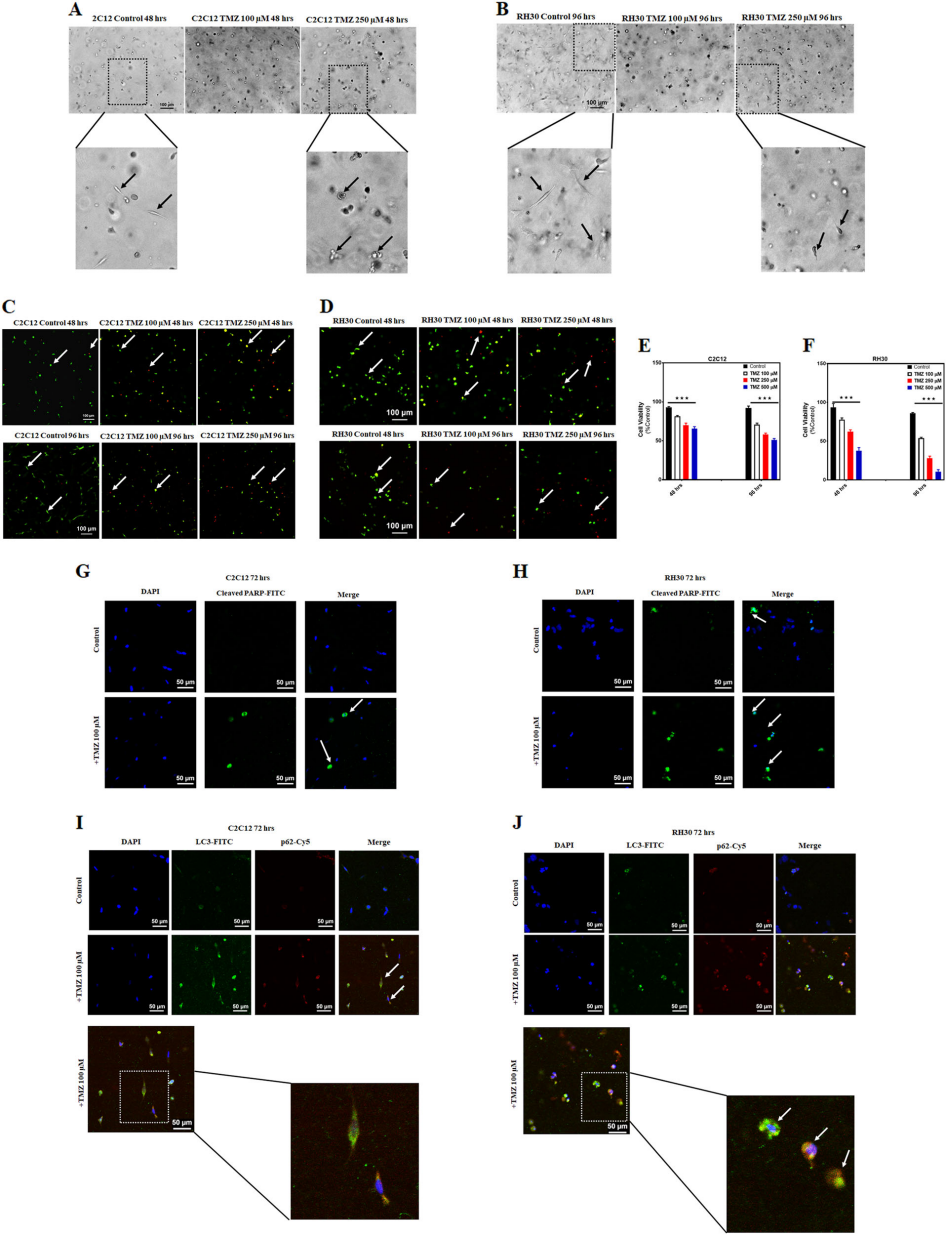
TMZ induces apoptosis and autophagy in C2C12 and RH30 3D culture. **a**, **b** Bright field image of C2C12 **a** and RH30 **b** 3D culture which shows the morphology of untreated and TMZ treated cells (100, 250 µM, 4 h) in 3D culture. **c**–**f** Viability assay was done by adding the live/dead solution to cells 48 and 96 h after treatment with TMZ (0–500 µM). Cells were incubated for 2 h in the dark at room temperature, rinsed twice with DPBS, and confocal microscopy was used to capture live/dead cell images in C2C12 **c** and RH30 **d** cells. **e**, **f** Quantification of live/dead assay was measured by calculating the ratio of live: total cells which showed a significant decrease in viability of C2C12 **e** and RH30 **f** cells treated with different concentrations of TMZ. The data showed TMZ significantly induces cell death in both C2C12 and RH30 cells (*P* < 0.001) while TMZ induces more cell death in RH30 compared to C2C12 cells. **g**, **h** IF labeling of C2C12 cells **g** and RH30 cells **h** by cleaved PARP following treatment with TMZ (100 µM, 72 h) increased number of cells with cleaved PARP in TMZ treated cells in comparison to control cells which is the hallmark of increase of apoptosis in these cells. **i**, **j** After treatment of C2C12 **i** and RH30 **j** cells with TMZ (100 µM, 72 h), cells were IF labeled with autophagosome markers, LC3 and P62. Data showed that TMZ increases LC3 puncta (green) which is localized with p62 compared to corresponding time match control, a hallmark of autophagy induction in C2C12 and RH30 3D culture

Three-dimensional (3D) cellular models have been extensively used to mimic the 3D microenvironment of cells and to study the effects of extracellular matrix as a barrier against drug diffusion^51,52^. We used 3D models of C2C12 and RH30 made of cell collagen disks (diameter = 5 mm and thickness = 1 mm µm) to evaluate the effect of TMZ. These disks were fabricated by mixing C2C12 and RH30 cells with collagen type I (3 mg/mL) at a final density of 2 × 10^6^ cells/mL and casting the disks in polydimethylsiloxane (PDMS) molds. After 24 h, cells were treated with TMZ (0–500 µM) for 48 and 96 h. Cell viability was evaluated by staining cells with green-fluorescent calcein-AM (live) and red-fluorescent ethidium homodimer-1 (dead) dyes (Fig. 4c, d). Our results showed a significant decrease in the number of live cells in TMZ-treated groups in both C2C12 and RH30 cells (*P* < 0.001), while TMZ induces more significant cell death in RH30 cells (Fig. 4e, f). Bright field images revealed significant changes in cell morphology of TMZ treated cells compared to control group in both C2C12 (Fig. 4a) and RH30 cells (Fig. 4b). Additionally, TMZ treatment also induced higher PARP cleavage compared to control in both C2C12 (Fig. 4g) and RH30 cells (Fig. 4h). Labeling of C2C12 and RH30 cells by using autophagy markers demonstrated an increase in the number of autophagosomes in TMZ-treated groups as compared to corresponding time match controls (Fig. 4i, j).

## Discussion

In the present study, we demonstrate that TMZ differentially induces apoptotic cell death in C2C12 and RH30 cells in both 2D and 3D culture models, and that the cell death is negatively regulated by autophagy. Interestingly, TMZ treatment does not change mitochondrial membrane potential in either RH30 or C2C12 cells, and the differential TMZ-induced apoptosis in C2C12 and RH30 cells cannot be entirely explained by changes in Bcl-2 anti-apoptotic (Bcl-2, Bcl-XL, and Mcl-1) and/or pro-apoptotic (Bax) protein expression.

There are currently a few therapeutic options available for patients with metastatic RMS^53^. To improve the therapeutic effect of chemotherapy in RMS, TMZ has recently received considerable interest as a factor for combination therapy because it is a well-tolerated oral alkylating agent with a broad spectrum of antitumor activity and relatively low toxicity^12,13^. Several Phase I trials suggest that using TMZ alone or in combination with other drugs is well tolerated in RMS when used in relapse settings^54^. The combination of irinotecan and TMZ, for instance, has shown synergistic antitumor activity against RMS^54–57^. Several studies have pointed out that autophagy acts as a pro-survival pathway which allows cancer cells to survive the existing stressed present in the tumor microenvironment^23,58,59^, such as the stresses caused by anticancer agents^60^. It has been found that autophagy and apoptosis have multiple direct and indirect interactions with each other^31,61,62^. In the present study, we observed that inhibition of autophagy could enhance the apoptotic effect of TMZ in C2C12 and RH30 cells; therefore, autophagy is a negative regulator of TMZ-induced apoptosis in both C2C12 and RH30 cells.

We have also determined that TMZ can activate apoptosis in a time and dose-dependent manner in RH30 cells. The enhancement of apoptosis was found to be associated with higher expression of histone H2A variant H2AX (data not shown). The phosphorylation of histone H2A variant H2AX at Ser139 is a marker of DNA double-strand breaks, a type of DNA damage that can lead to cell death, if unrepaired^63,64^. This mechanism of TMZ-induced apoptosis has been validated in previous studies in other cancer cell lines which is consistent with our results^11,65–67^. Our results demonstrated that intrinsic or mitochondrial pathway is involved in the induction of apoptosis after TMZ treatment in RH30 cells (significant increase in caspase-9 activation) while TMZ-induced apoptosis is not caspase-9 dependent in C2C12 cells. It has been reported that TMZ-induced apoptosis is caspase dependent in different models, including glioma cells^68–70^, and human glioma stem cells^71^. It has been previously reported that TMZ induces endoplasmic reticulum (ER)-stress with subsequent unfolded protein response in many cell models. We are currently investigating whether differences in the ER-stress response or the extrinsic apoptosis pathway underlies the differential responsiveness of C2C12 and RH30 cells to TMZ treatment^22,72^.

Mitochondrial Bcl-2 family proteins are considered to be one of the critical components in the regulation of apoptotic cell death^73–75^ and known to be essential for the apoptotic response in chemotherapy, emphasizing that these proteins are potential therapeutic targets^76,77^. Interestingly our results showed that TMZ-induced apoptosis is not associated with changes in Bcl-2, Bcl-XL, Mcl-1, and Bax expression in RH30 and C2C12 cell lines. It has been previously reported that TMZ-induced apoptosis decreases mitochondrial membrane potential in glioma cell line^78,79^ while our results showed that TMZ-induced apoptosis had not affected the mitochondrial membrane potential in both RH30 and C2C12 cells. This observation can be explained by our finding that TMZ has not affected the expression of anti-apoptotic (Bcl-2, Bcl-XL, and Mcl-1), and pro-apoptotic (Bax) Bcl-2 family proteins. In addition, our data show that inhibition of autophagy by using Bafilomycin-A1 increased TMZ-induced apoptosis in RH30 and C2C12 cell lines without affecting mitochondrial membrane potential in these cells

This pilot study has expanded our understanding of the cross-talk between apoptosis and autophagy controlling TMZ-induced apoptosis. It has been shown that the maintenance of cellular viability in RH30 cells is dependent on baseline autophagy^25^, while loss of ATG7 or Baf-A1 treatment can negatively regulate proliferation of RH30 cells^26,27^. We found that Baf-A1 inhibited baseline autophagy in RH30 and C2C12 cells and decreased their viability. More importantly, our study showed that the combination treatment of RH30 cells with Baf-A1 and TMZ has a synergic effect on TMZ-induced apoptosis compared to treatment with either TMZ or Baf-A1 alone. This suggests that autophagy has a survival role in RH30 and C2C12 cells treated with TMZ and the autophagy inhibitor enhances the anti-tumor effect of TMZ against RH30 cells by activation of apoptosis.

We have reproduced two-dimensional (2D) RH30 and C2C12 culture results in a 3D culture models (Fig. 4) which showed that TMZ induces higher apoptotic cell death in RH30 cells in the presence of autophagy activation in both cell models. These discoveries are an essential step for translational application towards developing new therapies for RMS. The findings in most cell-based assays are demonstrated in monolayer or suspension culture models, which may give false negative or positive results due to an artificial cellular environment. It also represents restricted significance for in vivo studies and often little value in foreseeing clinical effectiveness of both generally cytotoxic and molecularly targeted drugs^80^. 3D cultures have received more attention and become more accepted in the last few years as an essential strategic toolkit for developing new cancer therapy^80,81^. Furthermore, 3D in vitro culture systems mimic different aspects of human tumor tissue environment, therefore should be considered as an advanced model for routine anti-tumor drug testing^80^. In our current findings, we confirmed that 3D RMS culture can be used to dissect the role of TMZ in RMS therapy.

Our findings revealed that autophagy is a negative regulator of TMZ-induced apoptosis in both RH30 and C2C12 cells. In addition, we have shown that TMZ-induces MOMP and apoptosis that is not readily explained by changes in Bcl-2, Bcl-XL, Mcl-1, or Bax expression, and is not accompanied by changes in mitochondrial membrane potential.

## Materials and methods

### Drugs and reagents

Antibodies against human p62 (5114, 1:1000), Bax (5023, 1:1000), Bcl-2 (3498, 1:1000), Bcl-xl (2762,1:1000) Mcl-1 (94296, 1:1000) Bak (12105, 1:1000) Nix (12396, 1:1000) Beclin-1(3495, 1:1000), SQSTM1/p62 (D5L7G) (88588, 1:100 for ICC), LC3B (D11) XP® (3868, 1:100 for ICC), Cleaved PARP (Asp214) (D64E10) XP® (5625, 1:100 for ICC), and Bid (2002, 1:1000) were purchased from Cell Signaling Technology (Beverly, MA, USA); LC3 was purchased from Sigma-Aldrich (St. Louis, MO, USA); glyceraldehyde-3-phosphate-dehydrogenase (GAPDH) and Actin were purchased from Santa Cruz Biotechnology, Inc. (Dallas, TX, USA). Anti-rabbit IgG (whole molecule) and anti-mouse IgG (Fab specific) peroxidase-conjugated secondary antibodies were purchased from Sigma-Aldrich (St. Louis, MO, USA). Autophagy inhibitor Bafilomycin-A1 (Baf-A1), rabbit anti-human/mouse/rat LC3 (L8918, 1:3,000), anti-mouse IgG (A8924, 1:3000), Temozolomide, anti-rabbit IgG (A6154, 1:5000), propidium iodide (PI), and 3-(4,5-dimethyl-2-thiazolyl)-2,5-diphenyl-2H-tetrazolium bromide) (MTT) were purchased from Sigma-Aldrich Canada Co, Oakville, CA. Caspase-Glo®-9 assay was purchased from Promega (Toronto, ON, Canada). Enhanced chemiluminescence (ECL) prime regents (western blotting detection reagent) were purchased from Amersham-Pharmacia Biotech. Hyperfilm™ ECL was purchased from Fisher Scientific. Polydimethylsiloxane (PDMS) was purchased from Dow Corning®™, 5 mm diameter sterile biopsy punches were purchased from Stevens Company, 5 mg/mL bovine collagen type 1 with neutralized pH was purchased from Advanced BioMatrix. Penicillin-streptomycin (Pen-Strep, 10,000 units/mL penicillin and 10,000 µg/mL streptomycin) was purchased from Gibco®, fetal bovine serum (FBS) was purchased from Millipore Sigma. Secondary antibodies with conjugated fluorescence (Alexa Fluor® 488 AffiniPure Donkey Anti-Rabbit IgG, and Alexa Fluor® 647 AffiniPure Donkey Anti-Mouse IgG) were purchased the from Jackson ImmunoResearch Inc. DAPI (4’,6-Diamidino-2-Phenylindole, Dihydrochloride) was purchased from Thermo Fisher Scientific. IgG-free bovine serum albumin (BSA) was purchased from Jackson ImmunoResearch Inc. Live/dead viability kit was purchased from Millipore Sigma; formaldehyde 37% solution was purchased from VWR, Triton X-100 was purchased from Bio Basic Canada Inc. pCytochrome C-GFP was a gift from Douglas Green (Addgene plasmid # 41182)^40^.

### Cell lines and cell culture

Cell culture plastic ware, penicillin, and streptomycin were purchased from VWR (Toronto, ON, Canada). Cells were cultured in Roswell Park Memorial Institute (RPMI-1640) with l-glutamine and 25 mM HEPES (BioWhittaker; Cat #: 12-115Q) and Dulbecco’s Modified Eagle’s Medium (DMEM) (CORNING; Cat #: 50-003-PB) with 10% fetal bovine serum (FBS) (Gibco™; Cat #: 16000044). The human rhabdomyosarcoma cell line (RH30) [RC13, RMS 13, SJRH30] (ATCC® CRL¬ 2061™) (Human muscle cancer cells) and mouse muscle cell line (C2C12) (ATCC® CRL¬1772™) were used in this project. RH30 cell lines were cultured in (RPMI-1640) with l-glutamine and 25 mM HEPES media, and C2C12 cells were cultured in (DMEM) with high glucose media. Both media were supplemented with FBS (10%), penicillin (1%), and streptomycin (1%). Cells were grown to 35–40% confluency on a 100 mm cell culture plate, 6-well plates, and 96-well plates. Cells were maintained in a humidified incubator with 95% air and 5% CO_2_ at 37 °C and were passaged once every 2–3 days.

### Cell viability assay

The RH30 cells (30,000 cells/ml) and C2C12 (20,000 cells/ml) were seeded in 96-well plates and treated with different concentrations of TMZ in different time points (0–1000 μM, 0–96 h). Cells were also treated with various concentrations (0.1, 1, 2.5, 5, or 10 nM) of Baf-A1, an autophagy inhibitor, and cell viability was assessed after 48 and 72 h. In each time point, 20 µl of MTT 3-(4,5-dimethylthiazol-2-yl)-2,5-diphenyltetrazolium bromide (5 mg/ml), is the aqueous solubility of the reduced formazan product, was added into each well and incubated for 3 h. Then, media gently were removed from each well using pipette and 200 µl of solvent control (DMSO) was added to each well and mixed very well by pipetting to solubilize the MTT formazan. The plates were analyzed at a test wavelength of 570 nm on a plate reader, following a 20 min incubation at room temperature (RT)^82^.

### Immunoblotting

Western blot analysis was used to assess markers of apoptosis and autophagy in RH30 and C2C12 cells. We examined hallmarks of intrinsic or extrinsic apoptotic pathway and autophagy signaling pathways, while GAPDH or actin was used to normalize the results. After treatment, cells were collected, and protein extracts were made using NP-40 lysis buffer (20 mM Tris-HCl (pH 7.5), 0.5% Nonidet P-40, 0.5 mM PMSF, 100 µM β-glycerol 3-phosphate and 0.5% protease inhibitor cocktail). The extracts were kept in −20 °C until all extracts from different time points were collected. Samples were then sonicated in five times/five cycles using ultrasound sonicator, followed by centrifugation at 13,000×*g* for 10 min to collect the supernatant protein. Protein content was then determined via a Lowry protein assay, and protein samples were made. Prepared samples, of a volume between 15 and 20 µl, were heated at 90 °C for 5 min before loading into 10–15% polyacrylamide gels (depending on the molecular weight of the proteins). Additionally, 10 µl of a standard molecular weight marker (Thermo Fischer Scientific, ON, Canada) was loaded on each gel, as an approximate indicator of molecular protein weights. Proteins were immediately transferred under reducing conditions in transfer buffer (500 nM glycine, 50 mM Tris-HCl, and 20% methanol) to Immuno-Blot PVDF Membranes (Bio-Rad; #1620177), at RT and 100 volts for 2–2.5 h. Upon transferring completion, membranes were carefully transferred into 5% non-fat dried milk in 1X Tris-buffered saline containing Tween (TBS/0.025% tween 20; TBST) and placed on the shaker in the cold room overnight or RT for 2 h. Following blocking, membranes were incubated with the proper dilution of primary antibodies in 1% milk made in 1X TBST and kept in cold room (4 °C) overnight. Membranes were washed three times with 1X TBST (0.025% Tween) for 20 min, and membranes were incubated with secondary antibodies (HRP) for 2 h on the shaker at RT. Membranes were rewashed three times for 20 min and incubated with enhanced chemiluminescence (ECL) reagents (Amersham-Pharmacia Biotech) for 2–3 min. Autoradiography visualized the signals. Obtained protein bands were evaluated for changes in the autophagy and apoptosis signaling pathways. To assess even protein loading, membranes were incubated in milk 1% with primary antibodies against GAPDH or Actin overnight, washed three times and probed with a secondary antibody to visualize the signals. In the instances of re-probing of other proteins on the same membrane, blots were incubated with stripping solution containing 200 nM glycine, pH 2.5, 0.005 Tween 20 for 15 min at RT and followed the same instruction as after blocking for these blots^83,84^.

### Measurement of apoptosis by flow cytometry

Apoptotic cells were assessed by flow cytometry with propidium iodide (PI), using the Nicoletti method^85,86^. RH30 and C2C12 cells were treated with TMZ (100 μM, 72 h) in cells cultured in 12-well plates. In each time point cells were detached by EDTA buffer and centrifuged at 1500×*g* for 5 min at 4 °C. Then, cells were washed by PBS once. The cells were permeabilized and treated with a fluorescent dye that stains DNA quantitatively, using hypotonic PI lysis buffer (0.1% Triton X-100, 1% sodium citrate, 0.5 mg/ml RNase A, 40 μg/ml propidium iodide). Before flow cytometry analysis, cells were incubated for at least 1 h, at 4 °C, and in the dark to prevent photobleaching. The measurement was in red fluorescence (460 nm) for 10,000 cells. Flow cytometer was adequately calibrated to gate out debris accurately. Finally, after elimination of residual debris, the percentage of normal and apoptotic nuclei were estimated by analysis of the DNA histogram^86,87^. The nuclei of apoptotic cells were located on the left side of the G1 peak. Apoptotic nuclei have less DNA compared to nuclei of healthy G0/G1cells, causing an increase in sub-G1 section in the fluorescence histogram which can be applied to distinguish apoptotic cells in samples. In each sample, the sub-G1 peak was measured and statistically compared with other samples^86^. Annexin-V FITC and PI staining was performed according to manufacturer’s instructions (BD Biosciences 556547). Stained cells were analyzed on a Thermo Scientific Attune NxT flow cytometer with a 488 nm laser.

### Live cell imaging: LC3-GFP

GFP-LC3 is a specific marker for the occurrence of autophagosomes formation^88,89^. GFP-LC3 is the fusion of the green fluorescent protein (GFP) and LC3 and can behave similarly as endogenous LC3^90,91^. The GFP-LC3 is localized on the autophagosome membrane, and green punctate signals are observed^91^. To confirm TMZ-induced autophagy and autophagy flux inhibition through Baf-A1 (100 nM), cells were transfected with a green fluorescent protein plasmid called LC3-GFP (Addgene, #24920), a vector to visualize autophagosome formation in real time. C2C12s were transfected using JetPrime Polyplus reagent, while RH30 cell line was transfected using Qiagen’s Effectene reagent, as per manufacturer’s instructions. After 48 h of transfection, cells were treated with TMZ (60 h) and Baf-A1 (3 h before imaging). LysoTracker red staining (Molecular Probes™; LysoTracker® Red DND-99; L7528) was used to detect lysosomal activity and MitoTracker Red CMXRos at a concentration of 50 nM to detect active mitochondrial membrane potential. Cells were stained for 30 min in 37 °C incubator. Using this approach, instances, where LC3-GFP puncta co-localized with LysotTracker, were considered to be autophagic, while LC3-GFP co-localization with MitoTracker was interpreted as mitophagy^21^.

### Immunocytochemistry

For detection of autophagy flux, we used immunocytochemistry (ICC) in C2C12 and RH30 cells^31^. Briefly, RH30 cells were cultured on coverslips in 6-well plates in RPMI media with 10% FBS. The cells were then treated with TMZ (100 µM) or vehicle control and for 72 h. Four hours before time point Baf-A1 (100 nM) was added which already has been treated with TMZ. At the indicated time point, ICC was performed using the protocol described previously (Lysosomes were stained with LysoTracker red (Molecular Probes; 100 nM, 10 min) before fixation and permeabilization)^31,92^. GFP-LC3 punctuate co-localized with activated LysoTracker redd were identified as autophagic cells^92^.

### TMRM staining for mitochondrial membrane potential measurement

Healthy mitochondrial membranes hold the electrical potential difference between the exterior and interior of the cell, well known as membrane potential. This is an important process, which is linked to a multitude of mitochondrial function. Tetramethylrhodamine methyl ester (TMRM), a cell-permeant dye, can accumulate inside the healthy and active mitochondria with intact membrane potential which then becomes fluorescent^93,94^. TMRM fluorescent signal disappears when there is a loss of mitochondrial membrane potential. TMRM fluorescence can be detected with fluorescence microscopy which allows quantification of mitochondrial membrane potential. RH30 and C2C12 cells were cultured in 6-well plates (30,000 cells/ml) and treated with TMZ (100 μM, 60 h), Bafilomycin-A1 (Baf-A1, 4 nM) and TMZ/Baf-A1. By removing cellular growth media before staining, as well as well by washing C2C12s with phosphate buffered saline (1×, Hyclone), but not RH30, as RH30 cells can be easily detached, staining sensitivity was increased.

TMRM (Tetramethylrhodamine Methyl Ester Perchlorate), a mitochondrial membrane dye, and Hoechst, a nuclear dye, at concentrations of 100 nM and 10 μM, respectively, were diluted in media and added to cells for 30 min at 37 °C. Cells were imaged using an Olympus epifluorescence microscope. Fluorescence intensity was measured with ImageJ (NIH, Bethesda, MD, USA), minimum of 20 cells per condition. In each condition, fluorescence intensity for 20 cells (one by one) randomly was measured, and then intensity was averaged out for 20 cells and quantified^82^.

### Transmission electron microscopy (TEM)

TEM was used to evaluate autophagy activation in both cell lines following treatment with TMZ or Baf-A1 or combination. TEM imaging was performed according to a protocol described previously^21^. Briefly, RH30 and C2C12 cells were seeded in 100 mm plates (300,000 cells/dish) in RPMI and DMEM (high glucose) media, respectively, supplemented with 10% FBS. Cells were treated with TMZ and Baf-A1 and then collected using EDTA for cell detachment. Cells were centrifuged three times (1500×*g*) and then fixed (3% glutaraldehyde in PBS, pH 7.4) for 3 h at room temperature. Additionally, cells were treated with a post-fixation step using 1% osmium tetroxide in phosphate buffer for 2 h at room temperature, followed by an alcohol dehydration series, before embedding in Epon. TEM was performed with a Philips CM10, at 80 kV, on ultra-thin sections (100 nm on 200 mesh grids) 72 h after treatment. Cells were stained with uranyl acetate and counterstained with lead citrate for 3 min sequentially. Finally, grids were washed with water for 1 min and dried utterly to be ready for imaging. TEM was done to confirm the autophagy induction by TMZ and autophagy suppression by Baf-A1 in the cells.

### Three-dimensional (3D) culture

#### Fabrication of cell-loaded collagen disks

Disks of collagen with 5 mm diameter and 1 mm thickness loaded with 2 million cells/mL were used to perform 3D culture. The disks were made by curing the suspension of cells in collagen in PDMS holders placed in 12-well plates. To fabricate the PDMS holders, the base PDMS elastomer and the curing agent were mixed with a ratio of 10/1 and degassed using a vacuum chamber. The solution was then poured on a microscope slide and cured on a hot plate at 70 °C for 2 h to form a uniform 1 mm layer. The PDMS film was then cut to 15 mm square pieces, and a 5 mm hole was punched through them using a biopsy punch. To sterilize the PDMS holders, they were incubated in pure ethanol for 1 h and baked at 80 °C for 4 h to remove the ethanol. The PDMS holders were then placed in 12-well plates.

#### RH30 and C2C12 culturing in 3D

RH30 and C2C12 cells were cultured in a T75 culture flask in a 5% CO_2_ incubator at 37 °C, supplied with culture media (DMEM with 10% FBS and 0.5% Pen-Strep). The media was replaced every 24 h and the cells were collected at a confluency of 80%. To harvest cells, media were removed and cells were rinsed once with 4 mL of trypsin-EDTA, followed by 5 mL of trypsin-EDTA incubation for 5 min. Ten mililiters of media were  added to the cell suspension and centrifuged at 200×*g* for 5 min at 4 °C. The supernatant was removed, and the cells were re-suspended in fresh media and gently mixed with collagen at 4 °C to reach a final collagen concentration of 3 mg/mL and the cell density of 2 million cells/mL. Twenty microliters of the solution was added to each well in the PDMS holder and placed in the incubator for 45 min to cure the collagen. Then 2 mL of media was added to each well, and the cells were cultured overnight. Treatments were started after the incubation overnight. Two conditions were considered for the study which includes control (media) and TMZ (0, 100, 250, and 500 µM) treatment. The cells were treated for a total of 48, 72, and 96 h and then the viability and immunocytochemistry were performed. All experiments were performed in triplicates.

#### Live dead assay in 3D culture

Live/dead solution was prepared as per supplier’s protocol, where 5 µL of calcein AM and 20 µL of ethidium homodimer-1 were added to 10 mL of DPBS. After treatment, media were removed from the wells and live/dead solution was added followed by 2 h incubation at room temperature in the dark. Next, the solution was removed, and the wells were gently rinsed with DPBS twice. Confocal microscopy was performed right after the samples were stained. For quantifying viability, an image of each test case was considered to count the number of live and dead cells. The viability was quantified based on the ratio of the number of live cells to the total number of cells in each image.

#### 3D immunocytochemistry

Cells were fixed using 3.7% formaldehyde in DPBS, after removing media, for 40 min at room temperature. Next, the formaldehyde solution was removed, and the samples were washed with DPBS three times for 5 min each. Samples were then blocked using a solution of 5% BSA in DPBS with 0.3% Triton for 2 h at room temperature. Primary antibodies were diluted in DPBS with 1% BSA and 0.3% Triton, in a dilution factor of 1/300. The blocking solution was removed, and the primary antibody solutions were added to the samples and incubated overnight at 4 °C. LC3 and p62 were incubated simultaneously for co-staining and PARP were incubated separately. Next day, primary antibody solutions were removed, and samples were washed three times using DPBS for 5 min each. The secondary antibody solutions were made by diluting the secondary antibody in DPBS with 1% BSA and 0.3% Triton with a ratio of 1/300. Secondary antibodies were added to the samples and incubated for 2 h at room temperature in the dark. Secondary antibodies were later removed, and the solution of DAPI was added to the samples and incubated for 1 h at room temperature in the dark. Finally, DAPI solution was removed, and the samples were washed three times with DPBS for 5 min each. Confocal microscopy was performed right after the samples were stained.

### Statistical analysis

All results were presented as mean ± SD, and the differences between the groups were tested by one-way ANOVA or two-way ANOVA analysis (non-parametric, Brown–Forsythe test), using GraphPad Prism 7.0. The confidence interval in each analysis was 95%, and *P* < 0.05 was considered statistically significant.
